# Intra-Abdominal Compartment Syndrome Secondary to Massive Pelvic Müllerian Tumor

**DOI:** 10.7759/cureus.110521

**Published:** 2026-06-09

**Authors:** Sidra Shah, Tarunjog Singh Kalra, Durkho Atif, Umara Niazi

**Affiliations:** 1 General Internal Medicine, Russells Hall Hospital, Dudley, GBR

**Keywords:** abdominal compartment syndrome (acs), intra-abdominal hypertension, müllerian tumor, ovarian adenocarcinoma, rare gynecologic malignancy

## Abstract

Abdominal compartment syndrome (ACS) is a rare surgical emergency characterized by end-organ dysfunction resulting from sustained elevation of intra-abdominal pressure. Gynecological malignancies are an uncommon cause of ACS. We report the case of a 61-year-old woman who presented following an unwitnessed fall with hypotension, tachycardia, confusion, and marked abdominal distension. Initial laboratory investigations demonstrated severe acute kidney injury (AKI), hyperkalemia, anemia, and markedly elevated inflammatory markers. Computed tomography (CT) imaging revealed a massive septate cystic pelvic mass causing compression of the inferior vena cava (IVC) and displacement of bowel loops, with associated liver lesions and lymphadenopathy concerning for metastatic gynecological malignancy. Ongoing hemodynamic instability and evidence of end-organ dysfunction raised suspicion for ACS. The patient underwent emergency decompressive laparotomy with drainage of approximately 6 liters of cystic fluid, followed by total abdominal hysterectomy with bilateral salpingo-oophorectomy. Her postoperative course was complicated by persistent AKI, anemia, bilateral femoral vein thromboses, *Clostridioides difficile* infection, and lower-limb ulcers secondary to chemical burns. Initial histopathological findings were inconclusive; however, subsequent lymph node biopsy confirmed metastatic high-grade adenocarcinoma consistent with an ovarian or Müllerian primary malignancy. Due to advanced metastatic disease and profound frailty, the patient was deemed unsuitable for systemic oncological treatment and was transitioned to palliative care. She later died while receiving end-of-life support.

## Introduction

Compartment syndrome occurs when elevated pressure within a confined anatomical space compromises tissue perfusion and leads to organ dysfunction. Although commonly associated with extremity trauma, compartment syndrome can also affect the abdominal cavity [1]. Abdominal compartment syndrome (ACS) is defined as sustained elevation in intra-abdominal pressure associated with new organ dysfunction or failure [2]. According to the World Society of the Abdominal Compartment Syndrome, intra-abdominal hypertension is defined as intra-abdominal pressure greater than 12 mmHg, while ACS is defined as sustained intra-abdominal pressure greater than 20 mmHg with associated organ dysfunction [2].

ACS may be classified as primary or secondary. Primary ACS results from intra-abdominal or pelvic pathology such as trauma, hemorrhage, pancreatitis, or large intra-abdominal masses. Secondary ACS develops in the absence of primary abdominopelvic pathology and may occur in conditions such as sepsis, burns, or aggressive fluid resuscitation [3]. Elevated intra-abdominal pressure can impair venous return, reduce cardiac output, decrease renal perfusion, and compromise respiratory mechanics [4].

Gynecological malignancies are an uncommon cause of ACS. We describe a rare case of metastatic high-grade ovarian adenocarcinoma presenting with ACS and multi-organ dysfunction.

## Case presentation

A 61-year-old woman was brought to the Emergency Department (ED) following an unwitnessed fall at approximately 06:00. Her husband had last seen her seated at 04:00 and later found her on the floor beside her chair. She remained on the ground for approximately four hours before paramedic arrival. Her medical history was notable for progressive anorexia and significant unintentional weight loss over several months. She was a lifelong non-smoker and lived with her husband.

On initial assessment, paramedics documented hypotension and tachycardia and administered intravenous (IV) fluids and analgesia. In the ED, the patient appeared cachectic and confused, with a Glasgow Coma Scale (GCS) score of 14/15. Physical examination demonstrated marked abdominal distension with rigidity and absent bowel sounds. Bilateral lower-limb ulcers with features resembling pyoderma gangrenosum and bilateral pitting edema were also noted.

Initial laboratory investigations are summarized in Table 1.

**Table 1:** Initial bloods investigations.

Investigation	Result	Reference Range
Hemoglobin	70 g/L	120-160 g/L
White bloods cell count	13.9 ×10⁹/L	4-11 ×10⁹/L
C-reactive protein	296 mg/L	<5 mg/L
Potassium	6.8mmol/L	3.5-5.5mmol/L
Urea	39 mmol/L	2.5-7.8 mmol/L
Creatinine	361 µmol/L	45-90 µmol/L
Estimated glomerular filtration rate	11 mL/min	>60 mL/min
Pro-B-type natriuretuc peptide	6841 ng/L	<125 ng/L

Laboratory investigations demonstrated severe hyperkalemia, acute kidney injury (AKI), anemia, and significantly elevated inflammatory markers. The patient received IV piperacillin-tazobactam, hyperkalemia treatment, blood transfusion support, and fluid resuscitation. Due to abdominal rigidity and worsening hemodynamic instability, a surgical review was requested.

Initial computed tomography of the thorax, abdomen, and pelvis (CT TAP) could not be completed because of agitation. An abdominal X-ray (AXR) and erect chest radiograph demonstrated increased lower abdominal density and fecal loading, and the patient was commenced on laxatives and enemas.

Following stabilization, CT imaging of the abdomen and pelvis demonstrated a massive septate cystic pelvic mass measuring approximately 28 × 26 × 16 cm arising from the uterus or adnexa, causing marked compression of the inferior vena cava (IVC) and displacement of adjacent bowel loops (Figure 1). Multiple liver lesions and abdominal lymphadenopathy raised concern for metastatic gynecological malignancy.

**Figure 1:**
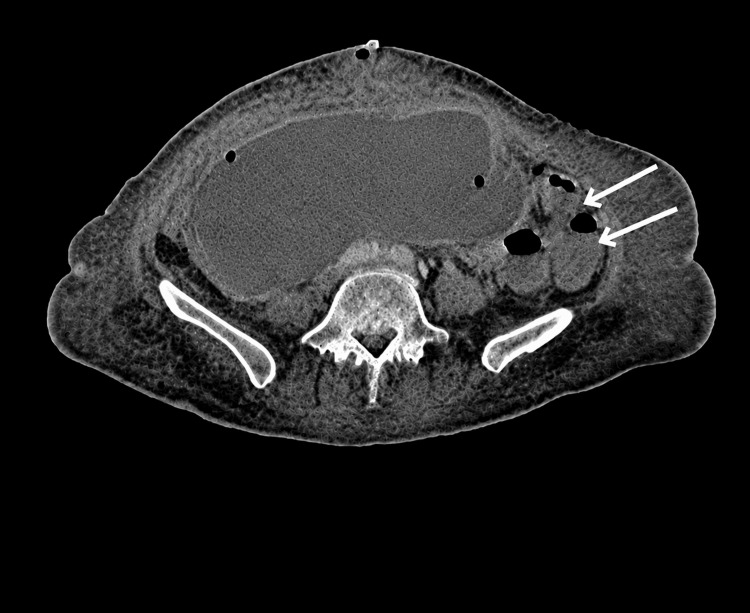
Axial contrast-enhanced CT scan of the abdomen and pelvis demonstrating a large septate cystic pelvic mass occupying the abdominal cavity and displacing adjacent bowel loops. The white arrows indicate displaced and compressed bowel loops containing intraluminal gas secondary to mass effect.

Given the patient’s progressive hemodynamic instability and associated end-organ dysfunction, particularly AKI, ACS was suspected. Urgent multidisciplinary involvement from gynecology, general surgery, and critical care teams was obtained.

Following multidisciplinary discussion, the patient underwent emergency midline laparotomy for abdominal decompression. Intraoperative findings revealed a large cystic and solid pelvic tumor extending to the xiphisternum and densely adherent to the bowel, pelvic organs, and peritoneal wall. Approximately 6 liters of cystic fluid were drained, followed by total abdominal hysterectomy with bilateral salpingo-oophorectomy and acquisition of omental and cyst wall biopsies. The patient developed intraoperative hypotension and hypothermia and was transferred to the intensive care unit (ICU) postoperatively.

Postoperatively, the patient experienced persistent AKI, anemia, and hypoalbuminemia requiring IV fluids, blood transfusions, and albumin replacement. Renal function gradually improved, with estimated glomerular filtration rate increasing to 88 mL/min following abdominal decompression.

Initial histopathological examination was inconclusive, demonstrating infarcted cyst wall tissue and degenerative cyst fluid without definitive malignancy. Omental biopsy was negative for malignancy. However, repeat imaging demonstrated a persistent pelvic mass, hepatic metastases, and right groin lymphadenopathy highly suggestive of metastatic ovarian carcinoma.

Doppler ultrasonography subsequently identified bilateral non-occlusive femoral deep vein thromboses (DVTs), likely related to malignancy-associated hypercoagulability and IVC compression. Therapeutic enoxaparin was commenced.

A gynecological multidisciplinary team (MDT) recommended image-guided biopsy of the right groin lymph node. Histopathological analysis demonstrated high-grade adenocarcinoma positive for CK7, CK20, PAX8, and estrogen receptor (ER), with proficient mismatch repair (pMMR) status and wild-type p53 expression, findings consistent with an ovarian or Müllerian primary malignancy. Molecular testing did not identify actionable mutations but supported an ovarian origin.

During admission, the patient developed several additional complications, including bilateral necrotic lower-limb ulcers later identified as chemical burns secondary to bee venom exposure, *Clostridioides difficile* infection requiring treatment with vancomycin and piperacillin-tazobactam, superficial wound dehiscence, recurrent hypotension, and worsening anemia.

Despite ongoing multidisciplinary management, the patient’s clinical condition progressively deteriorated with worsening frailty, persistent inflammation, poor oral intake, and declining functional status. Given the extent of metastatic disease, ongoing organ dysfunction, and poor physiological reserve, the oncology team concluded that she was unsuitable for systemic anti-cancer therapy.

Following discussion with the patient and her family, she was transitioned to palliative management and placed on a Gold Standards Framework (GSF) red pathway. The patient later died peacefully while receiving end-of-life care.

## Discussion

This case highlights a rare presentation of advanced ovarian malignancy complicated by abdominal compartment syndrome (ACS) and multi-organ dysfunction. Although ACS is well recognized in trauma and critical care settings, tumor-related ACS remains uncommon and can present substantial diagnostic and management challenges [3,5].

In this patient, the large pelvic mass produced a significant elevation in intra-abdominal pressure, resulting in impaired venous return from compression of the inferior vena cava (IVC), reduced cardiac output, hypotension, and renal hypoperfusion. The marked improvement in renal function following decompressive laparotomy strongly supports ACS as a major contributor to her acute clinical deterioration [4,6].

Ovarian cancer frequently presents at an advanced stage because early symptoms are often nonspecific. Abdominal distension, anorexia, and unintentional weight loss were prominent features in this patient. The presence of hepatic lesions and lymphadenopathy indicated disseminated disease at presentation [7,8]. Initial histopathological findings were nondiagnostic, likely due to extensive tumor infarction and necrosis. This case emphasizes the importance of repeat biopsy when radiological and clinical suspicion for malignancy remains high despite inconclusive pathology.

The patient also developed bilateral femoral DVTs, likely secondary to a combination of venous compression and malignancy-associated hypercoagulability. Venous thromboembolism is a recognized complication of advanced malignancy and contributes significantly to morbidity and mortality in oncology patients [9].

Management of ACS associated with malignancy is not standardized. Surgical decompression is generally reserved for patients with life-threatening organ dysfunction. Although decompressive surgery resulted in temporary physiological stabilization in this case, it did not alter the overall prognosis because of the extensive metastatic burden and poor functional status [5,10].

Current oncological management strategies place considerable emphasis on performance status when determining suitability for systemic treatment. In this patient, severe frailty, ongoing infection, recurrent hypotension, and progressive functional decline precluded chemotherapy or other active oncological interventions [11]. Consequently, palliative management was considered the most appropriate course of action.

## Conclusions

Advanced ovarian malignancy can rarely present with abdominal compartment syndrome and acute multi-organ dysfunction. Early recognition of ACS and timely surgical decompression may provide temporary physiological improvement; however, prognosis remains poor in the setting of advanced metastatic disease. This case also highlights the importance of repeat tissue sampling when initial histopathology is inconclusive and underscores the essential role of multidisciplinary and palliative care in the management of complex oncological emergencies.
